# Simultaneous Determination of α-Glucosidase Inhibitory Triterpenoids in *Psidium guajava* Using HPLC–DAD–ELSD and Pressurized Liquid Extraction

**DOI:** 10.3390/molecules25061278

**Published:** 2020-03-11

**Authors:** In-Cheng Chao, Ying Chen, Mei-Hua Gao, Li-Gen Lin, Xiao-Qi Zhang, Wen-Cai Ye, Qing-Wen Zhang

**Affiliations:** 1 State Key Laboratory of Quality Research in Chinese Medicine, Institute of Chinese Medical Sciences, University of Macau, Macao SAR 999078, China; yqzhou1986@gmail.com (I.-C.C.); cheny@sziit.edu.cn (Y.C.); LigenL@um.edu.mo (L.-G.L.); 2 Guangdong Institute for Drug Control, Guangzhou 510663, China; gaomeihua@gdidc.org.cn; 3 Institute of Traditional Chinese Medicine and Natural Products, Jinan University, Guangzhou 510632, China; chywc@aliyun.com

**Keywords:** *Psidium guajava*, HPLC–DAD–ELSD, pressurized liquid extraction, triterpenoids, corosolic acid, α-glucosidase inhibitory effect

## Abstract

*Psidium guajava*, a popular food and medicine dual purposes plant cultivated in tropical and subtropical regions, has been widely used as food crop and folk medicine, such as anti-diabetes agent, around the world. Triterpenoids have been considered as the major active ingredients of *P. guajava*. In the present study, a high-performance liquid chromatography coupled with diode array and evaporative light scattering detectors (HPLC–DAD–ELSD) method was developed for simultaneous determination of nine triterpenoids in *P. guajava*. Pressurized liquid extraction (PLE) was performed for sample preparation, and the analysis was achieved on a Cosmosil 5C18-MS-II (Nacalai Tesque, Kyoto, Japan) column eluted with gradient 0.1% aqueous formic acid-methanol system. The drift tube temperature of ELSD was set at 40 °C, and nitrogen flow-rate was at 1.6 L/min. All calibration curves for the analytes showed good linear regression (*R^2^* > 0.9992) within test ranges. The established method was validated for intra-day and inter-day precisions (RSDs < 5%) and accuracy (recovery 94.23–106.87%). The validated method was successfully applied to determinate nine triterpenoids in 15 samples from the leave or fruit of P. guajava. In addition, the α-glucosidase inhibition assay showed good α-glucosidase inhibition activity in almost all the determined triterpenoids. The present study suggested that triterpenoids should be the quality control markers for *P. guajava* and HPLC–DAD–ELSD was an effective tool for the quality control of *P. guajava*.

## 1. Introduction

*Psidium guajava,* an important *Myrtaceae* family plant cultivated in tropical and subtropical regions, is widely used as food crop and folk medicine around the world [1]. *P. guajava* has been planted in southern China, including Guangxi, Guangdong and Fujian provinces [2]. Current pharmacological studies revealed that *P. guajava* displayed a broad spectrum of pharmacologic activities, such as anti-diabetes [3], anti-cancer [4,5], anti-diarrhea [6], anti-oxidation [7], gastro [8] and liver protection [9] and anti-inflammation [10]. The leaves and fruits of *P. guajava* have been widely used for the treatment of diabetes and obesity in East Asia. The *P. guajava* leaf extract was approved as an antidiabetic agent in Korea [11]. Triterpenoids are the major components in the leaf of *P. guajava*. The total triterpenoids could ameliorate the development of diabetic peripheral neuropathy in rats, and improved insulin resistance in 3T3-L1 adipocytes [12,13]. Corosolic acid, the major triterpenoid in the leaf, is a very potent α-glucosidase inhibitor. Some herbs containing corosolic acid such as banaba have been used as antidiabetics [14]. Many corosolic derivatives also showed *α*-glucosidase inhibitory activities. The α-glucosidase, secreted in small intestine, hydrolyzes the carbohydrate into glucose before absorption into the blood stream. The inhibition of α-glucosidase retards the breakdown of carbohydrates and reduces the postprandial glycemia surge, which is beneficial for the treatment of diabetes [15].

Up to now, dozens of terpenoids have been isolated from *P. guajava* [16]. Among them, asiatic acid (**1**) [17], maslinic acid (**2**) [2], corosolic acid (**3**) [17], oleanolic acid (**8**) [18] and ursolic acid (**9**) [2] have been extensively investigated and shown to contribute to various pharmacological activities of *P. guajava* [19,20,21,22,23,24]. In addition, 3β-*O-cis-p-*coumaroyl-2α-hydroxy-olean-12-en-28-oic acid (**4**), 3β*-O-cis-p-*coumaroyl-2α-hydroxy-urs-12-en-28-oic acid (**5**), 3β*-O-trans-p-*coumaroyl-2α-hydroxy-olean-12-en-28-oic acid (**6**) and 3β*-O-trans-p*-coumaroyl-2*α*-hydroxy-urs-12-en-28-oic acid (jacoumaric acid) (**7**) were also identified from *P. guajava*; they are esters of maslinic acid or corosolic acid [25]. Our previous study showed that these compounds could be transformed into maslinic acid and corosolic acid by hydrochloric acid hydrolysis, which is a very cost-effective and time-saving method to produce corosolic acid and maslinic acid [26].

Several methods have been established to determine the active components of *P. guajava*. High-performance thin layer chromatography (HPTLC) was developed to quantify quercetin in *P. guajava* [27]. Flavonoids and flavonoid glycosides of *P. guajava* leaves were qualitatively analyzed by high-performance liquid chromatography coupled with diode array detector and mass spectrometry (HPLC–DAD–MS) [28]. An HPLC–DAD method with evaluation using PCA and Neural Network Analysis has been developed for simultaneous determination of 13 phenolic bioactive compounds in *P. guajava* [29]. An HPLC–PDA method was established for quantification of ursolic acid and oleanolic acid in the *P. guajava* [30]. Our previous research developed an HPLC method for quantification of corosolic acid and found the content of corosolic acid in leaf increased after acid treatment due to the hydrolysable corosolic acid derivatives. However, there is no method for simultaneous quantification of corosolic acid and its derivatives in *P. guajava*. Due to the absence of a chromophore, some compounds such as triterpenoids reveal poor UV absorption. Evaporative light scattering detector (ELSD) is a good choice for quantification of these compounds. Thus, HPLC coupled with DAD and ELSD method has been widely applied to analyze complex multiple constituents in Chinese medicine [31,32,33,34].

**Figure 1:**
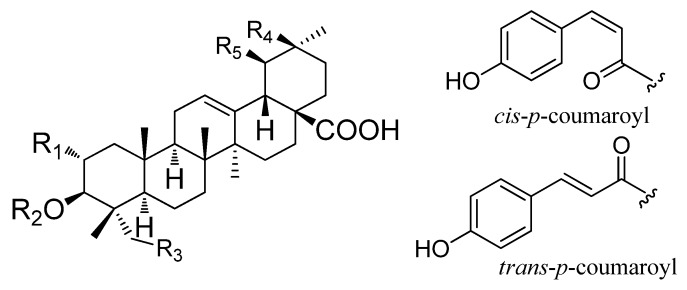
Chemical structures of nine triterpenoids from *P. guajava*.

In the present study, an HPLC-DAD-ELSD and pressurized liquid extraction (PLE) method was developed to simultaneously determine nine triterpenoids (Figure 1 and Table 1) in the leaves and fruits of *P. guajava* collected from different locations. In addition, the inhibitive effect of its leaves and fruits, as well as nine analytes on *α*-glucosidase were examined and compared.

## 2. Results and Discussion

### 2.1. Optimization of High-Performance Liquid Chromatography (HPLC) Conditions

**Figure 2:**
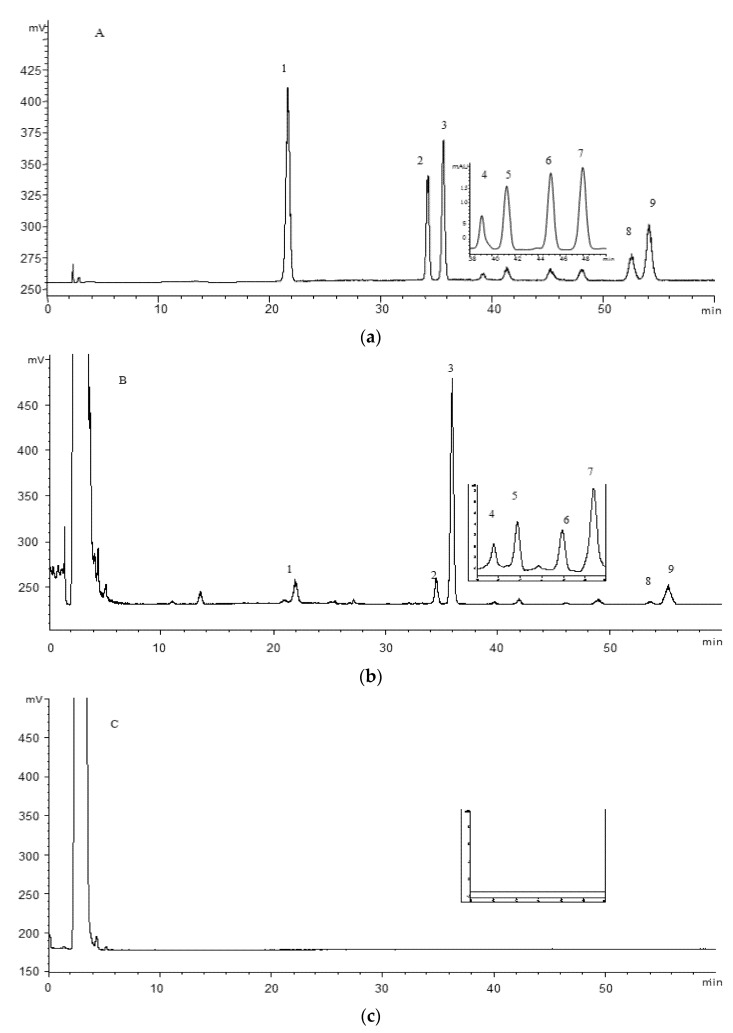
Representative chromatograms of mixed standards (**a**), the leaves (**b**) and fruits (**c**) of *P. guajava* detected by ELSD. The embedded chromatograms at 38–50 min are detected by the DAD at 310 nm: asiatic acid (**1**), maslinic acid (**2**), corosolic acid (**3**), 3β-*O*-cis-*p*-*coumaroyl*-2α-hydroxy-olean-12-en-28-oic acid (**4**), 3β-*O*-*cis*-*p*-*coumaroyl*-2α-hydroxy-urs-12-en-28-oic acid (**5**), 3β-*O*-*trans*-*p*-coumaroyl-2α-hydroxy-olean-12-en-28-oic acid (**6**), 3β-*O*-*trans*-*p*-*coumaroyl*-2α-hydroxy-urs-12-en-28-oic acid (**7**), oleanolic acid (**8**) and ursolic acid (**9**).

The optimization of HPLC conditions was carried out using sample PGL-2 (*P. guajava* leaves from Qingping). Several C18 and C8 columns from different companies and different gradient elution (acetonitrile-water and methanol-water) were tested and compared. C18 column in methanol-water system was more suitable for the separation. Formic acid as mobile phase modifiers could minimize the peak tailing, ameliorate the peak symmetry and improve resolution. We found that the compound **1**, **2**, **3**, **8** and **9** reveal very poor UV absorption because of the absence of a chromophore, while the compound **4**, **5**, **6** and **7** reveal good UV absorption because of the benzene ring. In the ELSD chromatogram, most triterpenoids were identified by comparison with the reference standards. Therefore, compound **1**, **2**, **3**, **8** and **9** were monitored by ELSD, and compound **4**, **5**, **6** and **7** were monitored by DAD at the optimum wavelength of 310 nm. Besides, the two important parameters of ELSD, drift tube temperature and nitrogen flow rate were also optimized using univariate analysis. In general, solvent evaporation is not completed at low temperature while the detector response is decreased at high temperature. Moreover, the low gas flow often results in spikes and noisy baseline; on the other hand, increasing the gas flow may result in a substantial decrease of the responses. Drift tube temperature was optimized systematically from 35 °C to 50 °C with a step of 5 °C and nitrogen flow rate from 1.3 to 1.8 L/min with step 0.1 L/min. As results, the five analytes obtain the best signal-to-noise ratio in ELSD signal with drift tube temperature of 40 °C and nitrogen flow rate of 1.6 L/min. The optimal HPLC–DAD–ELSD chromatograms are shown in Figure 2.

### 2.2. Optimization of Pressurized Liquid Extraction (PLE) Procedure

PLE is an extraction technique that employs solvent at elevated temperatures and pressures to extract analytes—mainly from solid samples. This extraction needs special equipment able to support both high temperature and pressure [35]. PLE is a green extraction method which could dramatically decrease the consumption of extraction time and solvent and had better repeatability compared to conventional extraction methods [36].

**Figure 3:**
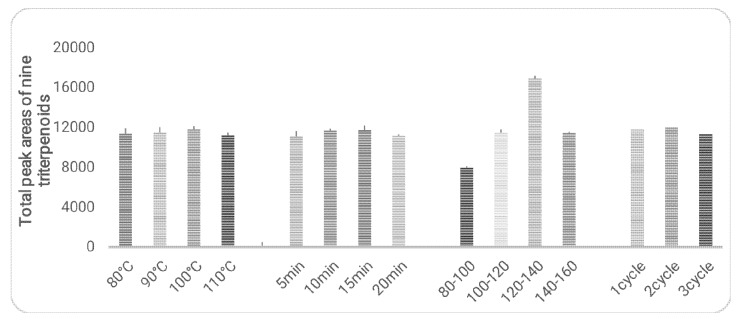
Influence of temperature, extraction duration, particle size and extraction cycles on extraction efficiency of PLE (*n* = 3).

The extraction procedures were optimized with sample PGL-2 (*P. guajava* from Qingping). The parameters including temperatures (80, 90, 100 and 110 °C) extraction durations (5, 10, 15 and 20 min), particle sizes (80–100, 100–120, 120–140 and 140–160 mesh) and extraction cycles (1, 2 and 3) were optimized using univariate analysis approach (Figure 3). The optimization results were shown in Figure 3, the total peak areas of nine triterpenoids were used as the marker to evaluate the extraction efficiency. The results suggested that particle size is the major factor that affects the extraction efficiency. No significant difference was observed from the other parameters. Taking time-saving into consideration as well as comparing the results of exhausted extraction, the best conditions of PLE extraction would be as follows: particle size, 120–140 mesh; temperature, 100 °C; static extraction duration, 10 min; number of extraction times, 1 cycle.

### 2.3. Method Validation

The developed HPLC–DAD–ELSD method was further validated for linearity, LOD, LOQ, inter- and intra-day precision and accuracy. All the nine analytes showed good linear with the correlation coefficients no less than 0.9992 (Table 2). The LOD and LOQ for the compound **1**, **2**, **3**, **8** and **9** which were monitored by ELSD were in the range 2.95–10.85 μg/mL and 9.84–36.15 μg/mL, respectively. While the LOD and LOQ for the compound **4**–**7**, which were monitored by DAD, were in the range 0.18–0.30 μg/mL and 0.61–1.01 μg/mL. The overall intra-day and inter-day variations of the analytes were less than 5% (Table 3). The sample stability test indicated that the sample was stable within at least 24 h, RSD of all the analytes were lesser than 3%. In addition, the accuracy of the method was satisfactory with recovery ranging from 94.23–106.87% (Table 4). All results suggested that this HPLC–DAD–ELSD method was reliable to quantitatively determine nine triterpenoids in *P. guajava.*

### 2.4. Quantitation of Triterpenoids in Fruit and Leaf of P. guajava

The developed HPLC–DAD–ELSD method was applied to quantify nine triterpenoids in fifteen *P. guajava* samples, including nine leaves and six fruits. The contents are shown in Table 5. It was found that asiatic acid (**1**), maslinic acid (**2**), corosolic acid (**3**) and ursolic acid (**9**) are main triterpenoids in the leaves of *P. guajava*, while oleanolic acid (**8**) was below the LOQ in the leaves. However, in this study, none of the triterpenoids could be detected in the tested fruit samples. In consideration of the report that several triterpenoids constituents isolated from fruits of *P. guajava* [37], it is plausible that triterpenoids are really presented in the fruits of *P. guajava*, but their contents are below the LOD. The results suggested that the leaves of *P. guajava* are one of potential plant resources rich in asiatic acid, maslinic acid, corosolic acid, ursolic acid and their derivatives.

### 2.5. Inhibition Activity of α-Glucosidase.

The effectiveness of *P. guajava* leaves and fruits, as well as nine triterpenoids, in inhibiting α-glucosidase activity were determined. As shown in Table 6, no inhibition activity was found in asiatic acid (**1**) and the fruits of *P. guajava*; the rest of analytical triterpenoids exhibited the diverse potential in α-glucosidase inhibition. Among these compounds, corosolic acid (**3**) showed the best inhibition activity with an IC50 of 1.33 µg/mL. However, the extract of *P. guajava* leaves exhibited more potential than any individual triterpenoid, indicating the synergistic effect of triterpenoids or other α-glucosidase inhibitors contained in *P. guajava* leaves.

## 3. Materials and Methods

### 3.1. Chemicals and Materials

Methanol and formic acid (HPLC grade) were purchased from Merck (Darmstadt, Germany). The ultra-pure water was purified using a Millipore Milli Q-Plus system (Millipore, Bedford, MA, USA). The α-glucosidase and *p*-nitrophenyl α-d-glucopyranoside were purchased from Sigma (St. Louis, USA). The enzymatic reaction results were detected on a SpectraMax M5 (Molecular Devices, San Jose, CA, USA).

The reference standards of triterpenoids **1**–**9** were previously isolated and identified by University of Jinan, and stored in the dark at 4 °C. The purities were determined to be greater than 98% by the normalization of the peak areas detected by HPLC–DAD–ELSD and confirmed by LC–MS, NMR spectroscopy.

The samples of *P. guajava* were purchased in local herbal stores or collected in Guangdong province, China, by our team members. All voucher specimens were deposited at the Institute of Chinese Medical Sciences, University of Macau, Macau, China.

### 3.2. Preparation of Standard Solutions

A mixed standard stock solution containing the nine reference compounds was prepared by dissolving them in methanol. The stock solution was consecutively diluted to obtain five gradient stock solutions. All the solutions were stored in a refrigerator at 4 °C until use and filtered through a 0.22 μm cellulose membrane before analysis.

### 3.3. Sample Preparation

A Dionex ASE 200 system (Dionex Corp., Sunnyvale, CA, USA) was used for sample preparation. Dried powder of *P. guajava* (0.50 g) was mixed with diatomaceous earth with a proportion of 1:1 and placed into an 11 mL stainless steel extraction cell; then the extraction was performed under the optimized conditions: 100% methanol; particle size: 120–140 mesh; temperature, 100 °C; static extraction time, 10 min; static cycle, 1 cycle; pressure: 1500 p.s.i.; flush volume, 40%. Then the extract was transferred to a 25 mL volumetric which was made up to its volume with 100% methanol, and filtered through a 0.45 µm Econofilter (Agilent Technologies, Santa Clara, CA, USA) before the HPLC analysis.

### 3.4. HPLC Analysis

An Agilent 1200 series HPLC system (Agilent Technologies, Santa Clara, CA, USA), equipped with on-line degasser, quaternary solvent delivery pump, auto-sampler, column compartment, diode array detector and Alltech 3300 evaporative light scattering detector (Grace, Deerfield, IL, USA) was used. A Cosmosil 5C18MS-II (4.6 mm × 250 mm, I.D., 5 μm) column was used for separation of the analytes. A gradient mobile phase consisted 0.1% formic acid in water (A) and methanol (B) and separation was achieved using the following gradient program: 1–18 min, 70% B; 18–20 min, 70–83% B; 20–60 min, 83% B; afterwards, washing column was performed with 100% B for 5 min and then return to the initial 70% B with 5 min post run time. The inject volume was 10 µL. The drift tube temperature of ELSD was set at 40 °C and the nitrogen flow rate was at 1.6 L/min. The gain ratio was at 8. Compound **4**, **5**, **6** and **7** were all monitored by DAD with the detection wavelength of 310 nm, while the rest of triterpenoids were detected by ELSD.

### 3.5. Method Validation

The developed method was validated in terms of calibration curve, sensitivity, precision, accuracy and stability.

For calibration curve construction, known amounts of nine triterpenoids were dissolved with absolute methanol and the stock solution was consecutively diluted to obtain five gradient stock solutions. Each concentration was analyzed in triplicate. Then, the calibration curves of **4**, **5**, **6** and **7** were constructed by direct plotting the peak area in DAD signal versus the concentration of each analyte, while the calibration curves of **1**, **2**, **3**, **8** and **9** were constructed by plotting the logarithmic of peak area in ELSD signal versus the logarithmic of the concentration of each analyte.

The sensitivity study was achieved by analyzing the limit of detection (LOD) and limit of quantification (LOQ) which were calculated as the concentration for each analyte with signal/noise ratio (S/N) at 3 and 10, respectively.

The precision of the method was determined by intra-day and inter-day repeatability. The intra-day repeatability was evaluated by extracting and analyzing sample PGL-2 (*P. guajava* leaves from Qingping) under the optimized extraction and chromatographic conditions, six replicates on the same day. For inter-day repeatability, the measurement was conducted one time a day for three consecutive days. The sample stability was tested by analyzing the sample of PGL-2 at 0, 4, 8, 12, 16, 20 and 24 h; the RSD of peak area of each component was recorded and compared.

A recovery test was used to evaluate the accuracy of the method. The recovery was determined by adding the investigated triterpenoids with high, middle and low levels to 0.25g sample PGL-9 (*P. guajava* leaves from Foshan) analyzed previously. The spiked samples were then extracted, processed, and quantified as above. Triplicates were carried out in order to compare their RSD. The recovery was calculated as the following equation:

Recovery (%) = (amount detected − amount original)/amount spiked × 100%(1)

### 3.6. Inhibition Assay of α-Glucosidase Activity

The inhibition assay of α-glucosidase was measured by the method as described previously [38,39,40,41]. Briefly, a total volume of 150 µL solution consisting of 100 µL phosphate buffer (0.1 M, pH 6.8), 20 µL α-glucosidase (2.4 unit/mg), 20 µL p-nitrophenyl α-d-glucopyranoside and 10 µL different concentration of test sample (12.5–500 µg/mL), was incubated for 30 min under the temperature of 37 °C, then the reaction was stopped by adding 80 µL 0.1 mM Na_2_CO_3_. The results were detected under the wavelength of 405 nm on a SpectraMax M5 (Molecular Devices, San Jose, CA, USA). The controlled sample used solvent instead of triterpenoids and the blank sample used buffer instead of α-glucosidase, respectively. The inhibition activity α-glucosidase was calculated by the following equation:

Inhibition (%) = [1 − (As − Asb)/(Ac − Acb)] × 100%(2)

Herein, As, Asb, Ac and Acb are absorption of the test sample, sample blank, control and control blank, respectively. Their half maximal inhibitory concentration (IC50) were calculated and compared.

## 4. Conclusions

In conclusion, a precise, accurate and reliable HPLC–DAD–ELSD and PLE method was successfully developed for simultaneous quantification of nine triterpenoids in leaves and fruits of P. guajava, which was helpful for the quality control of *P. guajava*. Our findings regarding α-glucosidase inhibition assay suggested that triterpenoids might be the active ingredients of *P. guajava* leaves for the treatment of diabetes.

## Figures and Tables

**Table 1:** Chemical structures of nine triterpenoids from *P. guajava*.

No	Chemical Name	R_1_	R_2_	R_3_	R_4_	R_5_
**1**	asiatic acid	OH	H	OH	H	CH_3_
**2**	maslinic acid	OH	H	H	CH_3_	H
**3**	corosolic acid	OH	H	H	H	CH_3_
**4**	3β-*O-cis-p-*coumaroyl-2α-hydroxy-olean-12-en-28-oic acid	OH	*cis-p-*coumaroyl	H	CH_3_	H
**5**	3β-*O-cis-p-*coumaroyl-2α-hydroxy-urs-12-en-28-oic acid	OH	*cis-p-*coumaroyl	H	H	CH_3_
**6**	3β-*O-trans-p-*coumaroyl-2α-hydroxy-olean-12-en-28-oic acid	OH	*trans-p-*coumaroyl	H	CH_3_	H
**7**	3β-*O-trans-p-*coumaroyl-2α-hydroxy-urs-12-en-28-oic acid	OH	*trans-p-*coumaroyl	H	H	CH_3_
**8**	oleanolic acid	H	H	H	CH_3_	H
**9**	ursolic acid	H	H	H	H	CH_3_

**Table 2:** Linear regression data, LOD and LOQ of the nine triterpenoids in *P. guajava.*

Analytes	Retention Time (min)	Calibration Curve ^a^	Test Range (ug/mL)	R^2^	LOD ^b^ (μg/mL)	LOQ ^c^ (μg/mL)
**1**	21.95	y = 1.39x + 4.46	3.03–48.40	0.9993	3.34	11.12
**2**	34.47	y = 1.52x + 4.76	3.43–54.80	0.9992	2.99	9.96
**3**	35.82	y = 1.50x + 4.60	7.75–124	0.9993	2.95	9.84
**4**	39.18	y = 8,165.82x -0.51	0.28–2.25	1.0000	0.30	1.01
**5**	41.48	y = 10,232.97x − 5.44	0.52–8.25	0.9998	0.25	0.85
**6**	45.75	y = 13,596.12x − 3.55	0.47–7.50	0.9998	0.18	0.61
**7**	48.30	y = 14,543.73x – 11.33	0.54–8.63	0.9999	0.23	0.77
**8**	52.49	y = 1.57x + 4.24	7.38–1.18	0.9993	10.85	36.15
**9**	54.27	y = 1.59x + 4.44	4.75–76.0	0.9992	9.76	32.53

^a^ y, peak area; x, concentration of the analytes (ug/mL); ^b^ Limit of detection (S/N = 3); ^c^ Limit of quantification (S/N = 10).

**Table 3:** Intra- and inter- day repeatability for determination of nine triterpenoids in *P. guajava.*

Analytes	Intra-Day (*n* = 6)		Inter-Day (*n* = 3)	
	Content	RSD (%) ^a^	Content	RSD (%)
**1**	4.30 ± 0.06	1.35	4.48 ± 0.20	4.50
**2**	3.6 ± 0.10	2.84	3.64 ± 0.01	0.39
**3**	19.27 ± 0.14	0.70	19.4 ± 0.86	4.44
**4**	0.7 ± 0.03	4.97	0.70 ± 0.01	1.81
**5**	1.25 ± 0.04	2.94	1.22 ± 0.03	2.18
**6**	0.77 ± 0.03	4.20	0.77 ± 0.02	2.70
**7**	1.63 ± 0.05	3.25	1.60 ± 0.03	1.90
**8**	ND ^b^	NA ^b^	ND	NA
**9**	4.26 ± 0.05	1.21	4.35 ± 0.17	3.96

^a^ RSD% = (S.D./mean) × 100%; ^b^ ND: Not detected; NA: Not adapted.

**Table 4:** Recoveries of the nine triterpenoids in *P. guajava.*

Analyte	Original (mg)	Spike (mg) ^a^	Found (mg) ^a^	Recovery (%) (*n* = 3)	RSD (%)
**1**	1.54	1.23	2.76	99.19	1.42
		1.60	3.25	106.87	1.02
		1.89	3.54	105.82	1.57
**2**	1.33	1.01	2.32	98.02	1.64
		1.38	2.74	102.17	3.37
		1.60	2.90	98.12	2.60
**3**	6.81	5.50	12.45	102.55	1.67
		6.80	13.70	101.32	1.24
		8.16	14.85	98.50	3.57
**4**	0.32	0.22	0.55	104.54	1.04
		0.33	0.66	103.03	1.97
		0.40	0.73	102.50	4.42
**5**	0.67	0.52	1.16	94.23	0.22
		0.62	1.27	96.77	4.33
		0.81	1.46	97.53	2.49
**6**	0.51	0.35	0.85	97.14	3.80
		0.52	1.05	103.84	2.74
		0.62	1.16	104.84	8.50
**7**	1.18	0.80	1.94	95.00	0.70
		1.18	2.39	102.50	1.15
		1.42	2.62	102.82	0.24
**8**	- ^b^	4.27	4.48	104.92	3.20
		4.98	5.32	106.83	1.87
		5.55	5.85	105.41	3.51
**9**	1.77	1.50	3.30	102.00	0.73
		1.79	3.58	101.69	1.32
		2.10	3.77	95.24	0.76

^a^ The data is presented as an average of three determinations; ^b^ Under the limit of quantitation.

**Table 5:** Contents (mg/g) of nine triterpenoids in fifteen *P. guajava* samples.

Samples No.	Location	Parts	1	2	3	4	5	6	7	8	9	Total
PGL-1	Zhanjiang	Leaves	2.50 ± 0.09	1.35 ± 0.07	7.05 ± 0.34	0.45 ± 0.1	1.01 ± 0.08	0.75 ± 0.03	1.78 ± 0.01	- ^a^	2.55 ± 0.07	17.94
PGL-2	Qingping1	Leaves	4.60 ± 0.07	3.85 ± 0.06	19.4 ± 0.07	0.74 ± 0.04	1.29 ± 0.04	0.83 ± 0.05	1.70 ± 0.05	-	5.30 ± 0.07	38.66
PGL-3	Conghua	Leaves	2.75 ± 0.07	2.15 ± 0.07	11.91 ± 0.13	0.46 ± 0.03	0.91 ± 0.02	0.62 ± 0.03	1.41 ± 0.08	-	3.28 ± 0.14	24.20
PGL-4	Qingping2	Leaves	3.34 ± 0.06	2.68 ± 0.09	14.17 ± 0.18	0.4 ± 0.01	0.81 ± 0.04	0.52 ± 0.01	1.11 ± 0.03	-	4.00 ± 0.08	27.83
PGL-5	Shunde	Leaves	3.26 ± 0.15	11.65 ± 0.24	5.95 ± 0.07	0.41 ± 0.02	0.77 ± 0.03	0.50 ± 0.03	1.21 ± 0.07	-	2.73 ± 0.16	16.74
PGL-6	Gaoming	Leaves	2.20 ± 0.09	0.91 ± 0.05	4.35 ± 0.04	0.27 ± 0.01	0.56 ± 0.04	0.34 ± 0.01	0.94 ± 0.02	-	2.41 ± 0.13	12.49
PGL-7	Macau1	Leaves	3.36 ± 0.05	2.31 ± 0.03	11.97 ± 0.17	0.77 ± 0.04	1.34 ± 0.05	0.80 ± 0.02	1.73 ± 0.06	-	4.83 ± 0.15	27.98
PGL-8	Guangzhou	Leaves	3.88 ± 0.10	2.83 ± 0.09	15.25 ± 0.32	0.78 ± 0.02	1.26 ± 0.06	0.74 ± 0.04	1.56 ± 0.03	-	4.89 ± 0.03	32.04
PGL-9	Foshan	Leaves	3.08 ± 0.19	2.67 ± 0.20	13.62 ± 0.30	0.63 ± 0.06	1.35 ± 0.05	1.03 ± 0.02	2.21 ± 0.03	-	3.54 ± 0.19	30.12
PGF-1	Macau1	Fruits	-	-	-	-	-	-	-	-	-	-
PGF-2	Gaoming	Fruits	-	-	-	-	-	-	-	-	-	-
PGF-3	Macau2	Fruits	-	-	-	-	-	-	-	-	-	-
PGF-4	Macau3	Fruits	-	-	-	-	-	-	-	-	-	-
PGF-5	Guangzhou	Fruits	-	-	-	-	-	-	-	-	-	-
PGF-6	Zhuhai	Fruits	-	-	-	-	-	-	-	-	-	-

^a^ Under the limit of quantitation.

**Table 6:** IC50 of nine triterpenoids and the methanol extracts of the leaves and fruits of *P. guajava* against *α*-glucosidase.

No.	IC50 (*n* = 3) against α-Glucosidase (µg/mL) *
**1**	NI
**2**	3.82 ± 0.03
**3**	1.33 ± 0.11
**4**	2.25 ± 0.28
**5**	1.54 ± 0.15
**6**	1.93 ± 0.14
**7**	2.12 ± 0.15
**8**	3.40 ± 0.28
**9**	4.35 ± 0.30
PGL	0.13 ± 0.00
PGF	NI

* All data are presented as Mean ± SE; NI: No Inhibition.
